# From Blame to Learning: The Evolution of the London Protocol for Patient Safety

**DOI:** 10.3390/healthcare13162003

**Published:** 2025-08-14

**Authors:** Francesco De Micco, Gianmarco Di Palma, Vittoradolfo Tambone, Roberto Scendoni

**Affiliations:** 1 Research Unit of Bioethics and Humanities, Department of Medicine and Surgery, Università Campus Bio-Medico di Roma, 00128 Roma, Italy; f.demicco@policlinicocampus.it (F.D.M.); v.tambone@unicampus.it (V.T.); 2 Operative Research Unit of Department of Clinical Affairs, Fondazione Policlinico Universitario Campus Bio-Medico, 00128 Roma, Italy; 3 Department of Law, Institute of Legal Medicine, University of Macerata, 62100 Macerata, Italy; r.scendoni@unimc.it

**Keywords:** patient safety, clinical risk management, systemic investigation protocol

## Abstract

Over the past two decades, patient safety and clinical risk management have become strategic priorities for healthcare systems worldwide. In this context, the London Protocol has emerged as one of the most influential methodologies for investigating adverse events through a systemic, non-punitive lens. The 2024 edition, curated by Vincent, Adams, Bellandi, and colleagues, represents a significant evolution of the original 2004 framework. It integrates recent advancements in safety science, human factors, and digital health, while placing a stronger emphasis on resilience, proactive learning, and stakeholder engagement. This article critically examines the structure, key principles, and innovations of the London Protocol 2024, highlighting its departure from incident-centered analysis toward a broader understanding of both failures and successes. The protocol encourages fewer but more in-depth investigations, producing actionable and sustainable recommendations rather than generic reports. It also underscores the importance of involving patients and families as active partners in safety processes, recognizing their unique perspectives on communication, care pathways, and system failures. Beyond its strengths—holistic analysis, multidisciplinary collaboration, and cultural openness—the systemic approach presents challenges, including methodological complexity, resource requirements, and cultural resistance in blame-oriented environments. This paper discusses these limitations and explores how leadership, staff engagement, and digital technologies (including artificial intelligence) can help overcome them. Ultimately, the London Protocol 2024 emerges not only as a methodological tool but as a catalyst for cultural transformation, fostering healthcare systems that are safer, more resilient, and committed to continuous learning.

## 1. Introduction

Over the last two decades, patient safety and clinical risk management have been among the central issues of global health policies, as a consequence of the growth in the complexity of care systems and the growing interest in the impact of adverse events [1]. The World Health Organization (WHO) has recently identified patient safety as a strategic priority for the quality of care, underlining the importance of a prevention-oriented approach and learning from errors [2]. Clinical errors and near misses are now known to be fine learning tools, capable of driving changes in organizational processes and improving the resilience and capacity to respond of healthcare systems [3,4].

In this context, the London Protocol has become one of the most applicable methodological tools for the analysis of clinical adverse events [5]. Put forward in 2004, the protocol has helped spread a view that goes beyond the search for “individual blame”, making room for the investigation of root causes and latent conditions that culminate in the production of errors. This approach is closely linked to the concept of “just culture”, which aims to create environments where healthcare personnel feel safe to report errors and key issues without fear of blame, thus fostering continuous improvement [6,7].

The new edition of the London Protocol (2024), edited by Vincent, Adams, Bellandi et al., represents a significant improvement over the original edition [8]. It welcomes the latest developments in safety sciences, human factors, and healthcare technology, including digital systems and artificial intelligence. Among the main innovations suggested are greater patient and family engagement, as main actors of the analysis and prevention of adverse events; appreciation for good practices and care successes, as well as errors, to identify factors promoting system resilience; greater psychological and organizational attention following an event, with healthcare personnel being recognized as “second victims” and with the need for adequate emotional and professional support; and asking to conduct fewer but more in-depth analyses in order to make specific, quantifiable, and sustainable suggestions, to prevent the risk of generating repetitive reports with little operational impact.

This updated version incorporates the systemic approach based on three main elements: the identification of care management problems (CMPs); the assessment of the presence or absence of defenses and barriers; and the consideration of eight levels of contributing factors (patient, individual, task, team, work environment, information and technology systems, organization and culture, and institutional context).

In this article, we want to outline the principles and innovations underpinning the London Protocol 2024 compared to the original 2004 version. We seek to determine how the protocol has been modified in line with the development of healthcare systems, safety sciences, human factors, and digital technologies. Another aim is to analyze the strengths and weaknesses of the systemic approach, with particular reference to its application in the Italian and European healthcare contexts. This allows us to establish how useful the model really is in optimizing care processes while also highlighting the most important problems that may arise in its application. The article also seeks to respond to the practical repercussions of incorporating the protocol within clinical risk management and patient safety programs. In particular, the role of patients, families, and healthcare professionals’ active participation—one currently at the center of adverse event prevention strategies—will be treated in detail. Finally, future prospects will be outlined, with a focus on the potential contribution of emerging technologies and artificial intelligence to clinical incident investigation and error prevention. In this manner, it is hoped to provide an updated and critical overview of the London Protocol, with a consideration of its potential to improve the safety culture of contemporary healthcare systems. This article is presented in the format of a narrative review, based on a critical conceptual analysis of both primary and secondary literature that pertains to the London Protocol, with the exact aim of situating its 2024 revision within contemporary patient safety frameworks. The articles read here were obtained in non-systematic ways from related journals in electronic repositories such as PubMed, Scopus, and Web of Science using keywords ‘London Protocol’, ‘patient safety’, ‘clinical risk management’, and ‘systemic approach’. The highest priority was given to peer-reviewed articles, institutional reports, and official documents published between 2004 and 2025.

## 2. The London Protocol 2024: Structure and Objectives

The London Protocol 2024 represents a significant overhaul of one of the most well-established approaches to systemic analysis of adverse events in healthcare. Hoping to support professionals and organizations in clinical incident investigations, the protocol is founded upon a crucial presupposition: errors are not typically the result of a single individual’s action but the culmination of a rich interplay of determinants linked—organizational, technological, environmental, and human. According to this vision, every unfavorable event is always an opportunity not only to know what occurred but also why it happened and under what systemic conditions its occurrence was possible.

The 2024 edition proposes a more focused and less bureaucratic system, calling for selective and thorough investigations instead of wide, superficial ones. The aim is not to produce a high volume of reports but to create concrete, actionable, and durable recommendations that can be translated into effective organizational reform. In this sense, the protocol is not just a reconstruction of history but also a learning and prevention tool, building the capability of the healthcare system to anticipate risk and build organizational resilience.

In terms of organizational structure, the London Protocol 2024 is built on three pillars:

Identification of problems in care management: the initial phase of analysis is focused on describing which aspects of care failed to function as intended, including gaps and deviations from planned standards.

Evaluation of defenses and barriers: this involves a review of currently present safety measures, i.e., a consideration of which barriers were effective, which were not, and which did not exist at all.

Analysis of error-contributing factors: the protocol utilizes a reference framework organized in eight layers of error-contributing factors, ranging from patient factors and individual performance to team factors, organizational issues, and institutional context. The multilayer organization enables capturing the complexity of interdependencies among people, technologies, and processes.

Among the most important developments of the 2024 edition is the greater emphasis on patients’ and families’ involvement as, respectively, important witnesses and requisite collaborators in the process of review. Their perspective generally provides insights complementary to those derived from healthcare providers, rounding out the analysis and rendering recommendations more pertinent. Moreover, the new protocol aims for the examination not only of adverse events, but of cases where the system has successfully prevented harm, with a goal to assess what mechanisms are responsible for resilience.

The new approach belongs to a contemporary understanding of safety that moves beyond blame and personal error in an effort to create a “just culture” founded on transparency, learning, and continuous development. The London Protocol 2024 is, in this sense, not only an investigative method but a driver of cultural progress that generates active prevention and deliberate management of risk (Table 1).

## 3. Practical Implications for Healthcare Systems

The London Protocol 2024 summons healthcare systems to a fundamental rethink of how they approach safety and risk. It is not simply a tool for investigating adverse events but a framework for a cultural and operational transformation. At its center is the recognition that safety does not spring from individual skill alone, nor from the strict following of procedures in a mechanistic fashion, but from the symphonic coordination between people, processes, technologies, and environments. The protocol encourages organizations to see incidents, near misses, and even good outcomes as windows into the way their systems function, providing a privileged glimpse into how care is delivered and where it could be better.

Operationally, putting the London Protocol 2024 into practice means moving beyond the traditional, reactive model of safety. Health organizations have been accustomed to examining adverse events after their occurrence, perhaps too often with the early notion of finding out what went wrong and who was at fault. This approach, while sometimes unavoidable, risks boiling down intricate events into linear narratives of error and blame [5,6,9,10,11]. The protocol revision suggests a broader and more nuanced way of thinking: rather than looking for single perpetrators, it asks teams to reflect on the layers of contributing factors, the circumstances of care provision, and the way that barriers and defenses succeed or fail. It also asks for reflection on what goes right in daily practice. By defining the conditions under which care may be safely delivered—e.g., effective teamwork, adaptive problem-solving, and robust system design—organizations can learn not only from failures but also from successes [12,13].

This shift toward a proactive mindset is not merely theoretical. Practically speaking, it will mean that safety will need to become part of the daily texture of clinical practice [14]. Safety rounds, multidisciplinary debriefs, and constant surveillance of near misses are no longer to be viewed as exceptional activities but as normal parts of how care is provided. In most hospitals, for example, formal daily briefings in which personnel have the chance to discuss problems as well as good experiences are proving themselves to be powerful mechanisms for building resilience [15,16,17]. The London Protocol 2024 reinforces this by emphasizing that safety is a journey and not a destination of ongoing learning, adaptation, and improvement.

The operationalization of the London Protocol in existing quality and safety programs also has practical effects. The majority of healthcare organizations are already conducting clinical audits, morbidity and mortality rounds, and root cause analyses [18,19]. However, such processes usually fall into predictable rhythms, producing formulaic reports not pursued to action [20]. The London Protocol offers a structured and systematic methodology complementary to these tools, interpreting events in their broader context in a consistent way. Instead of carrying out numerous superficial inquiries, organizations ought to be encouraged to prioritize depth over numbers and to focus on a few cases with the greatest potential for organizational learning. This avoids the fatigue and cynicism that may arise when incident reviews are perceived as bureaucratic exercises with minimal practical impact [21,22,23].

Practical experience supports this approach. In regions such as Tuscany, the application of the London Protocol has led to more accurate and helpful interventions [24]. For example, when a cluster of prescribing errors was identified, rather than attributing blame to individual physicians or nurses, analysis demonstrated that the major contributing factors were poorly designed electronic prescribing systems and inadequate communication at handover of care. As a consequence, the organization implemented redesigned software interfaces and standardized handover protocols. These alterations, which were concrete and long-lasting, led to measurable patient safety gains. Similar outcomes have been experienced by other healthcare systems, such as the United Kingdom NHS trusts, through the prioritization of fewer, more in-depth investigations instead of attempting to investigate each incident with the same intensity [25,26].

Successful application of the London Protocol requires skilled and multidisciplinary teams. No single investigator, regardless of experience, can expect to cover the range of modern healthcare systems. Teams need to be capable of bringing together different kinds of expertise, for example, clinicians, risk managers, human factors experts, and technical experts. The teams need to be educated not only in the mechanics of investigation but also in the principles of safety science. They need to find out about factors such as latent conditions, systemic defenses, and the interaction of human behavior and organizational structures. No less vital is the participation of frontline staff who are directly involved in the care processes under scrutiny. Their perspectives are often crucial to an understanding of the realities of daily practice—the workarounds, the pressures, the areas where safety margins come to be stretched.

It is not always simple to get the frontline staff involved, though. It requires a climate of trust and psychological safety. If staff believe that openness will lead to blame or punishment, then they will not give the information that is most useful for learning. The London Protocol 2024 stresses the importance of creating an environment where honesty is promoted and where the goal is to understand and improve systems, rather than punish individuals. Developing this culture is not straightforward and takes persistent leadership commitment, clear messaging, and visible modeling of just culture principles [26,27].

One of the most dramatic innovations in the 2024 edition is the active involvement of patients and families in incident reviews. This represents a significant departure from usual practice, where patients were often excluded from post-event analyses [28]. The new protocol recognizes that patients and families are not passive recipients of care but active agents whose experience and observations can shed light on the conditions leading to adverse events. Families can witness subtle breakdowns in communication or inconsistencies in plans of care that are opaque to clinical staff. By including their perspective, investigations can be rendered more complete, more accurate, and ultimately more effective.

Including patients and families also builds trust and transparency. By being open about the fact that they are open to hearing from and learning from those most immediately affected, organizations demonstrate accountability and a genuine commitment to improvement. This transparency can heal relationships damaged by adverse events and can enhance the reputation of the healthcare system as a whole [29].

A second critical aspect of the London Protocol 2024 is its requirement for providing meaningful and actionable recommendations. Too frequently, the output of an incident investigation is a report containing abstract or overly general recommendations—calling for “better communication” or “more training”—that are never translated into practice or evaluated [30]. The updated protocol avoids this pitfall by requiring that recommendations be specific, achievable, and budgetable. For instance, rather than merely suggesting that communication must be improved, an inquiry may specifically recommend implementing a formal communication tool such as SBAR, restructuring shift handover procedures, or adding simulation-based training to enhance teamwork during stressful situations.

Leadership is also key to these recommendations becoming a reality. Senior leaders must invest the necessary resources—time, personnel, and budget—to implement the interventions suggested. Leaders must establish mechanisms for monitoring progress, measuring outcomes, and holding teams accountable. Without this leadership commitment, even the most penetrating analysis risks being consigned to an academic exercise with little traction in the real world.

The advent of digital technologies and artificial intelligence (AI) adds another layer of complexity to incident analysis. Electronic health records, clinical decision support systems, and AI-driven diagnostic tools are now integral parts of healthcare provision. While they may reduce certain types of human error, they also carry novel vulnerabilities with them, e.g., algorithmic bias, data entry errors, or over-reliance on automated recommendations [31,32,33]. The London Protocol 2024 is sensitive to these challenges and requests that organizations examine both the technical and human aspects of technology use. If, for example, an AI diagnostic tool produces an incorrect diagnosis, the investigation needs to go beyond the algorithm itself to its integration into clinical pathways, the training provided to staff, and the steps taken to ensure critical appraisal of automated results.

Technology can also be a powerful ally in protocol rollout, as AI and data analysis software can help identify trends in incident reports, bring new hazards to light, and monitor the effectiveness of interventions over time. However, such tools must be used carefully, with a clear understanding of their limitations as well as potential unintended consequences [34].

Another crucial aspect is the well-being of healthcare staff, who have sometimes been referred to as “second victims” when they are involved in adverse events. The psychological and emotional effects of this can be profound, leading to stress, anxiety, and even burnout [35]. The London Protocol 2024 highlights that organizations have not only a duty to investigate incidents but also to look after the staff involved. Formal support programs, peer counseling, and facilitated debriefings can help staff process their experiences, learn from them, and still deliver high-quality care. Such support is not only an ethical necessity but a practical one as well: when staff are supported rather than blamed, they will be more willing to engage freely in reporting and learning processes [36].

Most challenging but with potentially transformative implications is the London Protocol 2024’s emphasis on building a just and learning culture. This cultural shift means progressing from punitive reactions to errors and building an environment where the overriding priority is to understand and improve systems. Leaders have a basic part to play in modeling this mindset, encouraging open communication, and ensuring that safety is viewed as a collective responsibility. This involves putting safety conversations into daily clinical and managerial routine, celebrating success but also analyzing failure, and encouraging staff at all levels to feel comfortable speaking up when they have concerns about risk [37].

Adhering to the London Protocol also has resource implications. High-quality investigations require time, expertise, and sometimes external facilitation. For trusts already under financial and staffing pressure, this is a challenge. Yet, the payoff from taking a long-term approach to systemic safety analysis is substantial. Through identifying and addressing underlying causes of harm, organizations can reduce adverse events, save themselves costly litigation, and win greater patient trust. And, in a world where the public’s expectations of healthcare are rising, the reputational benefits of being seen as an open, learning organization are increasingly valuable.

Finally, while UK-based in its origins, the London Protocol is universally applicable in its principles. The 2024 edition is drafted to be adaptable in different cultural and organizational settings. From big university hospitals to small community centers, in academic or community settings, the philosophy is the same: safety is a product of systems, not individuals, and requires continuous learning and improvement. Local adaptation is necessary, of course. In hierarchical organizations, effort may be necessary to allow junior staff to speak up during investigations. In low-resource settings, there may be a necessity to focus on a few high-priority cases or to simplify the data collection and analysis.

Lastly, the London Protocol 2024 is not just a method but a vision of what a learning healthcare system can be. By embracing its principles—systemic analysis, proactive learning, significant patient involvement, and a strong culture of trust—healthcare organizations can develop environments in which safety is not the result of chance but instead is actively created and recreated every day. It is a demanding framework, requiring commitment and perseverance, but its promise is significant: fewer preventable harms, better patient outcomes, and a workforce that feels sustained, appreciated, and capable of delivering care in a system that learns from every encounter (Table 2).

## 4. Strengths and Limitations of the Systemic Approach

The systems approach, as developed in the London Protocol 2024, is today one of the greatest success stories in the science of patient safety. Its formulation emerged from a recognition that traditional methods of analyzing adverse events, founded on individual blame, were unable to account for the nature of complex health systems today. For decades, errors were viewed as the direct consequence of incompetence or carelessness: the incompetent surgeon, the error-prone nurse who administered the wrong drug, or the technician who incorrectly calibrated an instrument [38]. While explanations are straightforward, they are typically not accurate or helpful. It does not prevent similar events from being repeated by punishing individuals. It is only by taking a step back from an individual’s actions, by asking why the system allowed the error to reach the patient, that we can actually learn and get better.

Since its first publication in 2004, the London Protocol has offered a completely different solution. It proposed that the query should not be “Who is to blame?” but “What conditions made this error possible?” Such an apparently simple shift in focus engages a far more nuanced level of understanding of adverse events. Examining an event is a matter of investigating the broader organizational and system forces behind it—communication breakdowns, process design flaws, incorrect levels of staffing, or shortage of technology and infrastructure.

It also involves analyzing the institutional and cultural environment that shapes workers’ caregiving patterns.” The aim is not to simply recognize errors but to understand the environment in which they occurred, in order to enable effective and sustainable changes. The 2024 London Protocol builds on these principles and refines them further, incorporating the idea that enhanced safety cannot rely on a single focus on what goes wrong but equally on an understanding of what goes right. Every day, thousands of complex tasks are accomplished without injuring anyone. Why? How does the system manage to function, even under stress or with a scarcity of resources?

This is the mindset that fits the Safety-II model, and it is one that stresses watching success as well as failure. It shifts the narrative from avoiding individual mistakes to enhancing the resilience and flexibility of the entire system [39]. One of the strongest aspects of the systemic approach is its holistic emphasis. An error can never be the result of an individual circumstance; it is the result of a series of connected circumstances and events [40]. Let us consider a medication error. A surface-level evaluation might argue that the nurse simply “was not paying attention”. The systemic approach considers more. Was the drug label hard to decipher? Was the electronic prescribing system poorly designed to yield confusing warnings? Was the nurse working an inordinately extended shift exhausted?

Were routine double-checking protocols missing or employed sporadically? Through examining these causal factors, the analysis moves beyond blame and identifies actionable changes that can render the system safer in the ensemble. Another key strength of the systemic approach is its ability to learn. Each bad event, and indeed each near miss, becomes an opportunity to learn about the process of care and to improve it [41,42].

The 2024 London Protocol gives special attention to this affirmative approach, inviting healthcare organizations not only to examine failures but to learn from situations in which harm was prevented. By discovering what barriers or defenses were effective, organizations can replicate and sustain those protections and build an even stronger system. Another feature of this approach is cooperation. Bad events are rarely the result of one person’s action, and their examination cannot be performed by one individual. The systemic approach relies on multidisciplinary collaboration, including physicians, nurses, pharmacists, clinical engineers, risk managers, and increasingly patients and families. Patient involvement, emphasized in the 2024 edition, reflects a profound culture shift.

Patients and carers can bring in useful different viewpoints on communication errors, delays, or misunderstandings that are unlikely to be picked up by professionals. By providing an avenue for the voices of directly impacted people to be heard, the inquiry not only becomes more valid and rich but also builds trust and openness—a foundation of a mature safety culture.

Despite these clear advantages, the London Protocol 2024 also presents several limitations and implementation challenges. One of the most significant weaknesses of the method is its methodological complexity. A complete systemic analysis needs specialized human factors, ergonomics, and risk management knowledge. It also consumes time and resources dedicated to it.

No healthcare organization may have well-trained teams that are capable of using the method properly. In some cases, analyses are performed by ad hoc teams with minimal experience, resulting in superficial reports and generic recommendations. Another concern is advice implementation. Even if an analysis has been conducted properly, the implementation of findings into actual improvements is difficult. Recommendations should not be too broad, lack clear accountability, or be unrealistic in the context of the organization’s constraints [43].

Having no strong leadership and adequate resources, even the best-intentioned proposals risk remaining on paper. The London Protocol 2024 addresses this by demanding practical, workable, and measurable recommendations that focus on those solutions that can realistically be implemented and evaluated over the long term. Cultural resistance still presents a significant hindrance. Within many healthcare environments, there remains a culture of blame, where mistakes are seen to be a matter of individual failure rather than system failure. This prevents staff from coming forward to report incidents or contributing freely to analysis. A just culture in which people are comfortable speaking up without fear of retribution is necessary if the systemic approach is to work [44,45,46]. Establishing such a culture is not simple.

It requires constant effort, explicit leadership backing, and a change in the attitude of organizations toward accountability—not as a search for the guilty, but as a common responsibility to learn and improve.

Another limitation is the lack of measurability of outcomes. Unlike other quality improvement initiatives, the impacts of system analyses can be non-obvious at first glance. Reductions in adverse events take time to manifest, and it can be hard to demonstrate a direct relationship between a specific question and an overarching safety enhancement. This can lead to frustration or a lack of ongoing support, particularly in organizations where quick returns are desired [47].

Resource constraints are also an issue, especially for the smaller hospitals or health centers with limited budgets and staff. The time and staff required to conduct detailed analysis and institute the suggested changes are considerable, both of which may be in short supply. In such environments, the systemic approach must be adapted—attending fewer high-payoff events and pragmatic solutions that can still make a good impact without overburdening the organization. The London Protocol 2024, in particular, attempts to address many of these challenges. Its emphasis on less but more intensive analysis attempts to break out of the quality-over-quantity trap. Its emphasis on patient engagement and on taking care of healthcare workers as “second victims” acknowledges the human and emotional dimensions of safety. Its integration of digital technology and artificial intelligence into its design also reflects the nature of modern healthcare, in which new equipment can both simplify and complicate safety efforts. In terms of future outlook, the systemic approach will need to continue moving forward. Within one of the largest challenges lies a culture of ongoing learning. Procedures and instruments are only as effective as the culture of the individuals who implement them. Healthcare organizations will need to establish an organizational climate where reporting, reflection, and openness become business as usual.

New technologies, while offering powerful new ability to analyze data and predict risk, will also introduce new types of error and weakness [48]. The systemic approach must be capable of responding to these realities in a manner that establishes an effective understanding and control over the relationship between human and digital systems. In short, the systemic approach offers a robust and visionary framework for patient safety improvement. Its benefits—thorough analysis, learning mindset, interprofessional teamwork, and adaptability—make it an essential tool in modern healthcare. However, its pitfalls—cultural resistance, complexity, and implementation challenges—require ongoing effort, leadership, and resources to overcome.

The London Protocol 2024 is more than an investigative process for mistakes but a blueprint for an error-wise healthcare system that values openness, common accountability, and continuous improvement. By recognizing both its abilities and its failings, organizations are able to embark upon a model of care where safety is not a post-event reaction but an active and core component of everyday practice.

For comparison with other established patient safety models, the London Protocol 2024 is useful to evaluate its strengths and limitations objectively. The comparison enables the determination of the distinct features of the London Protocol—like its systemic design, human factors integration, and direct patient and family participation—while also accentuating areas of possible weakness when juxtaposed against competing models. Through examining models such as HFACS-Healthcare [49], whose taxonomic categorical classification of contributory factors, or the WHO Patient Safety Framework [2], taking a macro-policy and governance perspective, one can ascertain where the London Protocol is adding value and where utilization could be improved upon through complementary methods. This benchmarking not only situates the London Protocol in context within the broader architecture of patient safety strategies but also informs decision-making with respect to its optimal use and possible integration with other tools with a view to promoting increased effectiveness of safety questions and interventions (Table 3).

## 5. Conclusions

Patient safety and clinical risk management are today among the most pressing dilemmas for global healthcare systems. Over the last few decades, heightened sensitivity to the effects of adverse events encouraged governments, international organizations, and healthcare providers to rethink error prevention and investigation strategies [50]. In this sense, the London Protocol has played a key role, becoming one of the most recognized tools for systemic analysis of clinical occurrences and for building up a culture of learning free of blame.

The London Protocol 2024 is a natural, albeit unavoidable, evolution, designed to consider the intrinsic changes that have conditioned healthcare in recent times. The heightened complexity of healthcare organizations, the rise of digital technologies, the expanding reach of artificial intelligence, and the risks from disorganized care pathways all necessitate more refined and adaptable tools of analysis. Compared to the original 2004 publication, the new protocol not only elaborates its basic principles but also enlarges upon them, such as organizational resilience, finding successes as learning opportunities, and the active participation of patients and families as safety allies.

Arguably, the most revolutionary aspect of the 2024 edition is its ability to balance depth and usability. The focus is no longer on conducting as many investigations as possible—an approach that often generates a multitude of reports with limited operational impact—but on carefully selecting events for analysis to produce recommendations that are realistic, measurable, and sustainable. This is a more advanced vision of risk management, one that transcends the simple learning curve of failures but seeks to strengthen the processes of harm avoidance and expand the capacity of the system to react in a timely manner.

No less important is the renewed emphasis on the human factor, not as a zone of risk but as a determining asset. The 2024 edition emphasizes the importance of teamwork, communication, and collaboration, and also the importance of supporting healthcare professionals who are part of adverse events—so-called “second victims”. In a safety-oriented healthcare system, the psychological and emotional health of the staff cannot be separated from the quality of care. Similarly, the influence of organizational culture cannot be overemphasized. Unless it operates within an environment of trust, openness, and shared learning, no protocol—no matter how carefully crafted—can ever succeed completely.

The inquiries conducted under the London Protocol 2024 extend beyond reconstructing what happened; they are a vision of the future, identifying systemic vulnerabilities and proposing changes that, if implemented, can transform the very organization. In this manner, the protocol is more than a tool of a technical nature but a catalyst for cultural transformation, instilling a proactive approach to safety in which prevention is embedded in the everyday practice of care delivery.

Going forward, bridging the discussion between technology and human experience will become a necessary aspect. Artificial intelligence and digital technologies present unprecedented opportunities for early detection of risks, predictive analysis, and decision support. Along with them come new issues, such as over-algorithmic dependency, data interpretation complexity, and the potential for tech-based errors. With its emphasis on human interaction, technology, and the organizational system, the London Protocol 2024 provides a solid foundation for meeting those challenges, but they will require ongoing refinement and adaptation.

Overall, the London Protocol 2024 is so much more than a new methodology; it is a statement of intent. It invites healthcare organizations to view patient safety not as a destination but as an ongoing dynamic process of learning and improvement. Its strength lies in combining robust analysis, active engagement by everyone involved, including patients, and systemwide attention to resilience and sustainability. Used consistently and judiciously, the protocol can assist in building healthcare systems that are safer, more resilient, and better able to handle tomorrow’s demands.

## Figures and Tables

**Table 1:** Summary of key points—London Protocol 2024.

Area	Key Points
Purpose	Structured approach to analyze incidents and clinical pathways, focusing on what they reveal about the healthcare system, not just “root causes”. Aims to promote learning, prevention, and safety improvement
Core principles	Keep analysis separate from disciplinary procedures; involve patients and families; focus on both negative outcomes and successes; a systemic approach based on human and organizational factors.
2024 updates	Greater emphasis on patient/family engagement, physical and psychological impacts, long-term timeframe analysis, assessment of consequences and support needs, and more detailed guidance for report writing and recommendation formulation.
Theoretical framework	Reason’s organizational accident model, extended to include corrective measures and post-event support; use of Problems in care management instead of “error”.
Contributing factors	Eight levels: (1) patient, (2) individual (staff), (3) tasks/activities, (4) team, (5) work environment, (6) information systems and technology, (7) organizational/managerial/cultural factors, (8) institutional context.
Preparation	Decision to investigate based on severity, frequency, or learning potential; possibility of thematic/aggregate analyses; need to select the most relevant events.
Reviewer training	Clinical or safety management background; human factors expertise; practical training; ability to manage difficult conversations and emotions after an incident.
Leadership and support	Senior management commitment required: just culture, functional governance, reporting and learning systems, adequate resources
Analysis process	Reconstruct chronology, identify good practices and PGAs, assess defenses/barriers, identify contributing factors, assess organizational culture, use scientific evidence, and draft report.
Recommendations	Based on a critical review of standards and procedures; clear prioritization; development of actionable and monitorable plans; avoid stereotyped or unfeasible recommendations.
Applications	Suitable for complex and rapid investigations; for adverse events, near misses, and positive-outcome cases; applicable in hospitals, community care, mental health, prison healthcare, and social healthcare settings.

**Table 2:** Key differences between the London Protocol 2004 and 2024 editions.

Dimension	London Protocol 2004	London Protocol 2024
Analytical Focus	Primarily focused on investigating adverse events	Expanded to include analysis of positive outcomes and system resilience
Approach to Causation	Emphasis on root cause analysis and linear models of failure	Adopts a systemic, multilayered approach, including contributing factors at 8 levels
Stakeholder Involvement	Focused mainly on internal healthcare professionals	Emphasizes active involvement of patients and families as witnesses and co-analysts
Team Composition	Single or mono-professional investigative teams	Promotes multidisciplinary teams, including clinical, technical, human factors, and managerial experts
Psychological Safety	Limited attention to the emotional impact on staff	Recognizes healthcare workers as “second victims”, recommending emotional and professional support
Output of Investigations	Often general recommendations (e.g., “improve communication”)	Requires specific, actionable, and measurable recommendations
Cultural Orientation	Aligned with “just culture” principles but often applied within blame-prone settings	Strong push toward a learning and non-punitive culture
Use of Technology	Minimal reference to digital systems or AI	Integrates digital health, EHRs, and AI-based tools as both contributors and supports for analysis
Application Strategy	Encouraged widespread use across events	Advocates for fewer but deeper investigations, focused on high-impact cases

**Table 3:** Comparison of the London Protocol 2024, HFACS-Healthcare, and the WHO Patient Safety Framework.

Characteristic	London Protocol 2024	HFACS-Healthcare	WHO Patient Safety Framework
Primary objective	To conduct a systemic analysis of clinical incidents (with or without harm) in order to identify contributing factors and strengthen safety and organizational culture.	To systematically classify the causes of errors according to hierarchical levels of human and organizational factors.	To provide a strategic framework and operational tools to reduce clinical risk, promote a safety culture, and standardize safe practices.
Methodological approach	Narrative and factor-based analysis (eight levels of contributing factors), reconstruction of the patient pathway, and active involvement of patients and families.	Taxonomy with four levels (Active Errors, Preconditions, Unsafe Supervision, Organizational Factors), derived from Reason’s model.	Macro-systemic approach based on “Priority Action Areas” (e.g., leadership, data, training) and operational checklists.
Analytical structure	(1) Identification of good practices and problems in care management; (2) Analysis of defenses/barriers; (3) Identification of contributing factors; (4) Recommendations and action plan	(1) Active Errors; (2) Preconditions for errors; (3) Inadequate supervision; (4) Organizational factors.	(1) Leadership and governance; (2) Safety culture; (3) Reporting and learning systems; (4) Priority clinical interventions (e.g., surgical safety checklist).
Patient/family involvement	Central: active inclusion in investigations, immediate and long-term support	Not included in the original model, but can be incorporated through local adaptation.	Recommended as a guiding principle, but not structured as part of the analytical process.
Human factors	Fully integrated in the framework (individual, team, work environment, technology, organization, institutional context).	Primary focus: detailed analysis of errors and latent conditions related to human factors.	Considered broadly (training, workload, communication) but mainly at the policy level.
Applicability	Broad: hospital, community care, mental health, prison healthcare, home care; adaptable to complex events and near misses.	Clinical events in any setting, particularly useful for complex incidents involving multiple human interactions.	All levels of healthcare, especially for national or large-scale organizational programmes.
Typical output	Detailed report including chronology, contributing factors, prioritized recommendations, and monitored improvement plan.	Causal mapping and tree diagram, classification into standardized categories, useful for databases and trend analysis.	Guidelines, performance indicators, monitoring and evaluation tools.
Strengths	Depth of analysis, active stakeholder engagement, adaptability to different contexts and timeframes.	Taxonomic clarity, standardization enabling statistical analysis, and cross-case comparisons.	Global vision, alignment with international standards, strong institutional recognition.
Limitations	Requires time, advanced training, and strong organizational support to be effective.	May be overly rigid or focused on classification rather than on solution development.	Less operational-analytical detail for individual events, more strategic than investigative in nature.

## Data Availability

No new data were created or analyzed in this study. Data sharing is not applicable to this article.
